# DNA methylation of imprint control regions associated with Alzheimer’s disease in non-Hispanic Blacks and non-Hispanic Whites

**DOI:** 10.1186/s13148-024-01672-4

**Published:** 2024-04-25

**Authors:** Sebnem E. Cevik, David A. Skaar, Dereje D. Jima, Andy J. Liu, Truls Østbye, Heather E. Whitson, Randy L. Jirtle, Cathrine Hoyo, Antonio Planchart

**Affiliations:** 1 Toxicology Program, North Carolina State University, Raleigh, NC USA; 2 Center for Human Health and the Environment, North Carolina State University, Raleigh, NC USA; 3 Department of Biological Sciences, North Carolina State University, Raleigh, NC USA; 4 Bioinformatics Research Center, North Carolina State University, Raleigh, NC USA; 5 Department of Neurology, School of Medicine, Duke University, Durham, NC USA; 6 Department of Family Medicine and Community Health, Duke University, Durham, NC USA; 7 Department of Medicine, School of Medicine, Duke University, Durham, NC USA; 8 Duke Center for the Study of Aging and Human Development, Durham, NC USA; 9 Duke/UNC Alzheimer’s Disease Research Center (ADRC), Durham, NC USA

**Keywords:** Alzheimer’s disease, Epigenetics, Imprint control regions, DNA methylation, Computational analysis

## Abstract

Alzheimer’s disease (AD) prevalence is twice as high in non-Hispanic Blacks (NHBs) as in non-Hispanic Whites (NHWs). The objective of this study was to determine whether aberrant methylation at imprint control regions (ICRs) is associated with AD. Differentially methylated regions (DMRs) were bioinformatically identified from whole-genome bisulfite sequenced DNA derived from brain tissue of 9 AD (5 NHBs and 4 NHWs) and 8 controls (4 NHBs and 4 NHWs). We identified DMRs located within 120 regions defined as candidate ICRs in the human imprintome (https://genome.ucsc.edu/s/imprintome/hg38.AD.Brain_track). Eighty-one ICRs were differentially methylated in NHB-AD, and 27 ICRs were differentially methylated in NHW-AD, with two regions common to both populations that are proximal to the inflammasome gene, *NLRP1,* and a known imprinted gene, *MEST/MESTIT1*. These findings indicate that early developmental alterations in DNA methylation of regions regulating genomic imprinting may contribute to AD risk and that this epigenetic risk differs between NHBs and NHWs.

**Supplementary Information:**

The online version contains supplementary material available at 10.1186/s13148-024-01672-4.

## Introduction

More than six million Americans are affected by Alzheimer’s disease (AD) [1], which is the most common form of dementia (60% to 80% of cases) [2, 3], and it is now the sixth leading cause of death in the USA [3]. Additionally, AD places a tremendous burden not only on the patients, but also on the caregivers and the healthcare system. AD is a disease of cognitive changes, but also increases susceptibility to multiple comorbidities, including pneumonia, femur fractures, and increased mortality risk [3]. This neurodegenerative disease is associated with cell death and atrophy involving various brain regions, which progress along anatomically connected networks, starting in the entorhinal cortex and medial temporal lobes, and extending into the neocortex over time. Although it is now accepted that this neuropathology is characterized by the aggregation of extracellular amyloid-beta (Aβ) plaques and intracellular neurofibrillary tangles (NFTs) composed of the hyperphosphorylated tau protein [4], the etiologic factors contributing to AD are still largely unknown. Established risk factors for AD, including advanced age, familial history, genetics, history of head trauma, and cardiovascular diseases do not fully explain the formation of Aβ plaques and NFTs [1].

A modest number of genetic variants derived from hypothesis-driven and agnostic approaches have been associated with AD. For familial AD, which affects ~ 5% of cases, genetic risk factors include mutations in genes such as *apolipoprotein E* (*APOE*; Chr19) [5, 6], *amyloid precursor protein* (*APP*; Chr21) [7, 8], *presenilin 1* (*PSEN1*; Chr14) [7, 9], *presenilin 2* (*PSEN2*; Chr1) [7, 10–12], and *beta-site APP-cleaving enzyme 1* (*BACE1;* Chr11) [13, 14]. *APOE* contributes to both familial and sporadic AD, and has three variants, ε2, ε3, and ε4 [5, 15] of which ε4 is the most prevalent isoform found in AD cases [16]. These genetic risk factors are often associated with abnormal protein function (*i.e.,* proteinopathies), which is believed to play a significant role in familial AD [16].

However, up to 95% of the disease is estimated to be sporadic [16, 17]. Such cases share common neuropathological endpoints with familial AD, including Aβ plaque accumulation, NFTs, synaptic loss, excess inflammation, oxidative damage, and neuronal death [18]. While genetic factors, such as APOE variants, appear to influence sporadic AD through intricate interplay with each other and environmental influences, it is important to note that they are neither necessary nor sufficient for the development of AD [17]. This has led to the emerging hypothesis that environmental stressors accumulated over the life course contribute to the later development and progression of AD [19].

Globally, there is a slight geographic variation in AD prevalence [20]. Among Americans aged 65 years and older, the risk of AD is twofold higher in non-Hispanic Blacks (NHBs) and 1.5-fold higher in Hispanics compared to non-Hispanic Whites (NHWs) [1]. The causes for this disparate outcome are presently unclear. Although a single nucleotide polymorphism (rs115550680) of *ABCA7*, which regulates lipid transport [21], was recently associated with late-onset AD in a NHB population but not NHW or Hispanics [22], this known genetic variant does not fully explain the higher disease burden in NHBs. A plausible hypothesis to account for the elevated rates of disease in NHB and Hispanic populations is that environmental or life course stressors, such as migration and segregation [23], inadequate medical surveillance, and living in polluted environments, are more common in these populations and result in functional and enduring alterations in the epigenome [24, 25]. Thus, profiling epigenetic marks that link established risk factors to AD holds promise for early detection, and for identifying novel mechanistic pathways contributing to AD.

Epigenetic dysregulation, which can cause alterations in gene expression in response to environmental stressors, may cause long-term changes in molecular pathways contributing to AD. Indeed, it was recently documented that the average 5-methylcytosine level is decreased in the entorhinal cortex of individuals with AD compared to that in controls [26]. DNMT1, a critical factor in the maintenance of DNA methylation, and MeCP1/MBD2, components of the methylation complex, are also significantly decreased in the entorhinal cortex of AD individuals compared to controls [27]. Furthermore, brain-derived neurotrophic factor (*BDNF*), which functions in cortical neuron maintenance, has increased promoter CpG methylation in both AD brain tissue and blood [28], in support of similarities between methylation patterns in the blood and brain tissues of AD and other dementia patients [29, 30]. Nevertheless, the interpretation of these data is complicated by lack of replication, and the possibility that methylation levels may change between tissue/cell type and throughout life.

In epidemiological studies, DNA methylation is frequently identified in accessible peripheral blood [31–34], but because these epigenetic marks can differ between tissues and cell types, they do not always correlate with those from inaccessible cells of affected brain regions. Moreover, because epigenetic marks respond to various environmental cues throughout life, causality is difficult to discern. One exception to these issues is the repertoire of methylation marks controlling genomic imprinting—the human imprintome—which epigenetically regulates the expression of imprinted genes crucial to tissue development during the intrauterine period [35]. After fertilization, DNA methylation of imprint control regions (ICRs) in the primordial germ cells (PGCs) undergoes complete erasure, and after sex determination, these regions are remethylated in a sex-dependent manner. This time frame is a window of high susceptibility to epigenetic perturbations due to environmental exposures and stressors that can alter the methylation of these ICRs in PGCs [36, 37]. PGCs with aberrant methylation can then transfer altered gene expression to the next generation and because of mitotic heritability, this aberrant methylation is conserved in all cell types and tissues in the offspring resulting in altered health effects over the life course [38], and increased susceptibility in adulthood to diseases, such as AD.

Thus, the complete mapping of the human imprintome that is susceptible to environmentally influenced alterations is key to understanding the non-genetic factors in complex diseases [35]. It is also important to distinguish between the non-imprinted epigenetic-controlled regulatory sites, and the ICRs involved in regulating the parental-dependent expression of imprinted genes. DNA methylation patterns at non-imprinted sites are cell type-specific and can be responsive to environmental cues throughout life. In contrast, the inherited ICRs, or the somatic ICRs that occur at the stem cell stage of embryonic development, should have the same stable methylation status across all tissues throughout life, including peripheral blood cells and the brain [39]. As changes in the brain are likely to start decades before clinical symptoms of AD appear [40, 41], the purpose of this study was to use genome-wide approaches to comprehensively identify dysregulated ICRs [35] associated with AD that trace their origin to adverse events in early development. The consistency of imprinted methylation marks across tissues and cell types makes them attractive as early epigenetic biomarkers for AD obtainable from accessible tissues.

## Results

### Patient and sample characteristics

Characteristics of the 17 NHW and NHB individuals who donated the brain samples used in this study are shown in Additional file 1: Table S1. AD cadavers ranged in age from 63 years to > 89 years (median 84 years), and controls ranged in age from 56 to 87 years (median 74 years). All nine AD samples were obtained from the temporal cortex. Five of eight control brain samples were obtained from the temporal cortex while three were obtained from the cerebellum. The diagnosis of AD was made postmortem through a comprehensive neuropathologic evaluation (Additional file 1: Table S1). The brain tissues used in this study were obtained from the Joseph and Kathleen Bryan Brain Bank at Duke University, which has historical significance, as it contained brain samples that were instrumental in the discovery of the association between APOE-ε4 and late onset and sporadic Alzheimer's disease [5, 6].

### Association of ICRs with Alzheimer’s disease

**Fig. 1:**
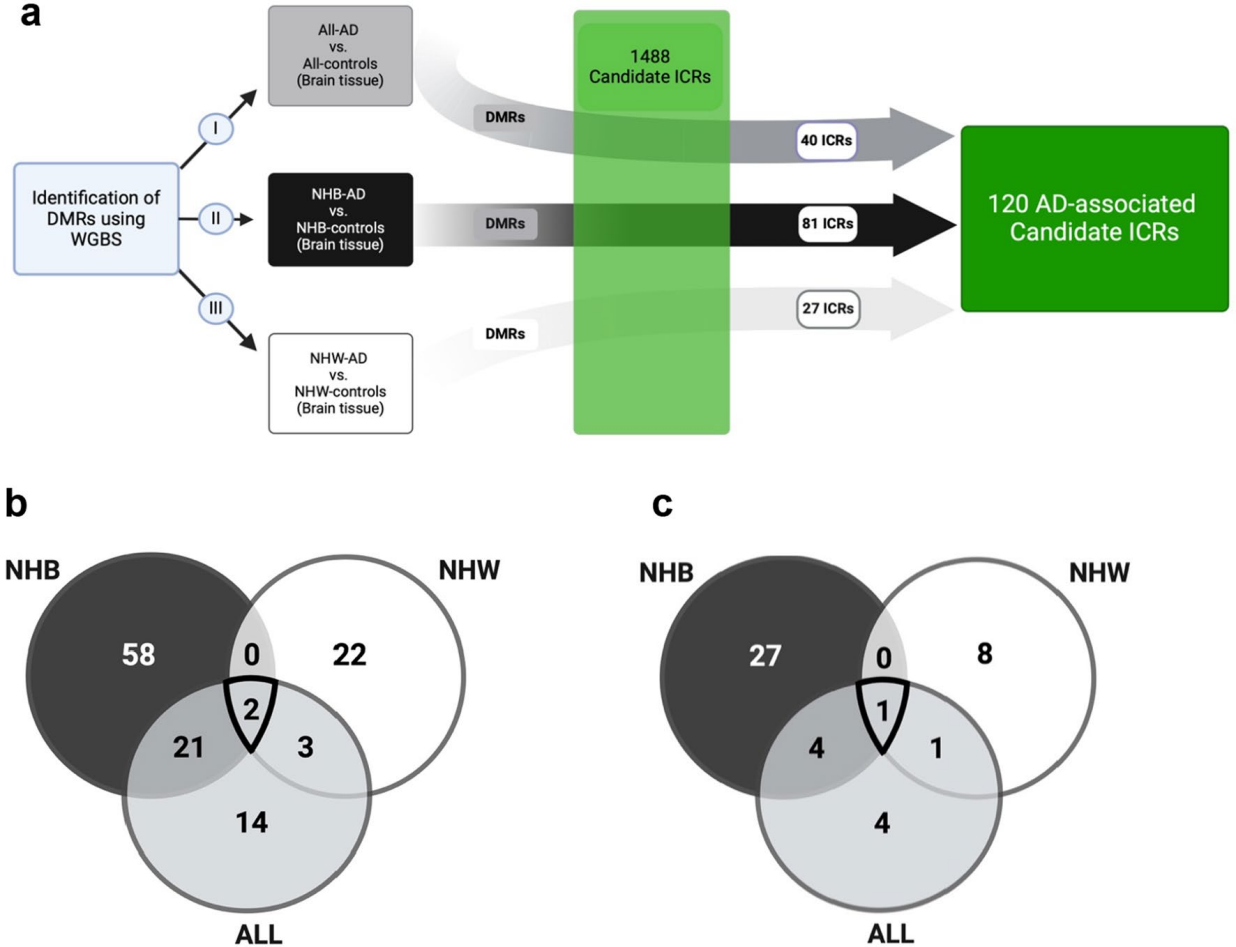
AD-associated candidate ICRs in NHBs and NHWs. **a** DMRs that differed in DNA methylation (≥ 10%) between AD cases and controls in NHBs and NHWs were determined by WGBS. **b** Venn diagram of ICRs from ALL [40], NHB [81] and NHW [27] when DNA methylation differed by ≥ 10% between AD cases and controls. **c** Venn diagram of ICRs from ALL [10], NHB [32] and NHW [10] when DNA methylation differed by ≥ 15% between AD cases and controls. Created with BioRender.com

Bisulfite conversion rate of AD and control samples taken from individuals of NHBs and NHWs showed a > 97% bisulfite conversion in all sample groups. Quality controls revealed no sequence duplication bias (Additional file 1: Fig. S1a), and sequence coverage between 15X and 36X (Additional file 1: Fig. S1b, c). We identified the CpG methylation ratio from replicate bam files for AD samples and controls. Later, differentially methylated regions (DMRs) were called using model-based analysis of bisulfite sequencing data criteria (MOABS, version 1.3.8.7) [42]. We performed three different analyses to identify AD-associated DMRs (Additional file 2: Table S2) using a 10% differential methylation threshold, a minimum read depth $\ge$ 7, and a maximum distance between consecutive CpG’s $\le$ 300, consistent with MOABS [42]. The resulting set of AD-related DMRs were analyzed against the 1488 candidate ICRs reported by our group [43], resulting in the identification of 120 candidate ICRs, including four of the 25 confirmed ICRs, that exhibit differential methylation in AD patients compared to controls (Fig. 1a, Table 1). Stratified by group, **I.** 40 (33.3%) differentially methylated ICRs are observed between all AD samples (n = 9) and controls (n = 8), **II.** 81 (67.5%) are observed between NHB-AD cases (n = 5) and controls (n = 4), and **III.** 27 (22.5%) are observed between NHW-AD cases (n = 4) and controls (n = 4). Interestingly, our results indicate that NHBs exhibit a threefold increase relative to NHWs in AD-associated differential methylation of regions postulated to be ICRs (Fig. 1a, Table 1). Alignment of AD-related DMRs and candidate ICRs can be accessed at https://genome.ucsc.edu/s/imprintome/hg38.AD.Brain_track.

**Table 1:** AD-associated DMRs overlapping with candidate ICRs

Number	ICR	ICR coordinates	DMR coordinates	Nearest transcript	Distance to closest gene (bp)	Race/ethnicity	%Parental methylation
1	ICR_17^	chr1:8117511–8117827	chr1:8117067–8117532	RPL7AP18	59,024	NHB	P
2	**ICR_20^ (A)**	**chr1:10682902**–**10683413**	chr1: 10682586– 10683160	CASZ1	0	NHB	P
	**ICR_20^ (B)**	**chr1:10682902**–**10683413**	chr1: 10682972– 10683160	CASZ1	0	ALL	P
3	**ICR_39**	**chr1:38210131**–**38210429**	chr1:38210102–38210802	LINC01343	0	NHB	P
4	ICR_54	chr1:112742974–112743310	chr1:112743018–112743108	NUTF2P4	4785	NHB	M**
5	**ICR_55^ (A)**	**chr1:116641652**–**116642769**	chr1:116642521–116642780	IGSF3	0	NHB	
	**ICR_55^ (B)**	**chr1:116641652**–**116642769**	chr1: 116642465–116642749	IGSF3	0	ALL	
6	ICR_67^&^	chr1:161442725–161442826	chr1:161442654–161442757	FCGR2A	62,604	NHB	
7	**ICR_78^ (A)**	**chr1:203076164**–**203076460**	chr1:203076065–203076495	PPFIA4	0	NHB	
	ICR_78^ (B)	chr1:203076164–203076460	chr1: 203076065 –203076234	PPFIA4	0	ALL	
8	**ICR_88**	**chr1:228619143**–**228619258**	chr1:228619100–228619407	RNA5S5	0	NHB	
9	**ICR_89^**^**&**^	**chr1:228620967**–**228621017**	chr1:228620811–228621043	RNA5S6	428	NHW	
10	**ICR_92^**	**chr1:228632865**–**228633148**	chr1:228632864–228633210	RNA5S11	116	NHB	
11	ICR_93^^&^	chr1:228635189–228635657	chr1:228635411–228635654	RNA5S12	200	NHW	
12	ICR_94	chr1:228636250–228636431	chr1:228636252–228636353	RNA5S13	663	NHW	
13	**ICR_106^**	**chr1:244729465**–**244729883**	chr1:244729523–244729966	DESI2	20,432	NHB	
14	ICR_116^	chr2:28342638–28342806	chr2:28342650–28342769	BABAM2	3737	NHB	P
15	**ICR_125 (A)**	**chr2:54289850**–**54290281**	chr2: 54289947 –54290188	ACYP2	0	NHB	M
	**ICR_125 (B)**	**chr2:54289850**–**54290281**	chr2: 54289947– 54290255	ACYP2	0	ALL	M
16	ICR_137^&^	chr2:105236991–105237239	chr2:105237149–105237194	GPR45	4504	ALL	M**
17	ICR_140^^&^	chr2:112433099–112433213	chr2:112433150–112433260	RGPD8	0	NHW	
18	**ICR_144**	**chr2:120526146**–**120526533**	chr2:120525904–120526415	LINC01101	59,797	NHB	P
19	ICR_163*^ (A)	chr2:181574336–181575348	chr2: 181574501 –181574918	CERKL	0	NHB	
	ICR_163*^ (B)	chr2:181574336–181575348	chr2: 181575328– 181575420	CERKL	0	NHB	
20	ICR_180^	chr2:230991059–230991241	chr2:230991094–230991158	SPATA3	4883	NHB	
21	ICR_188	chr2:241902453–241902725	chr2:241902629–241902778	LINC01237	0	NHB	M
22	ICR_203*^	chr3:46558604–46558922	chr3:46558570–46558679	LRRC2	0	ALL	M**
23	ICR_207	chr3:50481168–50481455	chr3:50481297–50481636	CACNA2D2	0	NHB	P
24	ICR_211 (A)	chr3:96776774–96777017	chr3:96776899–96777065	CDV3P1	0	NHB	
	ICR_211 (B)	chr3:96776774–96777017	chr3:96776927– 96777065	CDV3P1	0	ALL	
25	ICR_238*^^&^	chr4:1050914–1051080	chr4:1050995–1051010	RNF212	5168	ALL	
26	ICR_244	chr4:3702565–3703061	chr4:3703028–3703124	LINC02171	24,710	ALL	P
27	ICR_268	chr4:55158026–55158180	chr4:55158025–55158163	KDR	32,431	NHB	
28	**ICR_273^**	**chr4:81153935**–**81154202**	chr4:81153919–81154077	PRKG2	0	NHW	
29	ICR_281	chr4:152009424–152009915	chr4:152009643–152010206	RNA5SP169	37,811	ALL	P
30	ICR_287*^ (A)	**chr4:184097251**–**184097401**	chr4:184097176–184097474	ENPP6	0	NHB	M**
	ICR_287*^ (B)	chr4:184097251–184097401	chr4: 184097286 –184097326	ENPP6	0	ALL	M**
31	ICR_315	chr5:55178062–55178152	chr5:55177765–55178063	CDC20B	4885	NHB	M**
32	ICR_324	chr5:110894251–110894443	chr5:110894333–110894361	BCLAF1P1	51,830	NHB	M
33	ICR_326*^	chr5:136079156–136079563	chr5:136079562–136079640	TGFBI	15,338	NHB	M
34	**ICR_327^ (A)**	**chr5:136079902**–**136080957**	chr5:136080178–136081178	TGFBI	16,084	NHB	M**
	ICR_327^ (B)	chr5:136079902–136080957	chr5: 136080656– 136080693	TGFBI	16,084	ALL	M**
35	ICR_352	chr5:171319025–171319892	chr5:171318815–171319422	TLX3	6886	NHB	P
36	**ICR_367^ (A)**	**chr6:10099407**–**10099822**	chr6:10098961–10099504	OFCC1	0	NHB	
	ICR_367^ (B)	chr6:10099407–10099822	chr6:10099389– 10099472	OFCC1	0	ALL	
37	ICR_423*^	chr6:170423032–170423404	chr6:170423063–170423130	FAM120B	15,284	NHB	
38	ICR_434*^ (A)	**chr7:2019844**–**2020175**	chr7:2019610–2020099	MAD1L1	0	NHB	
	ICR_434*^ (B)	chr7:2019844–2020175	chr7: 2019100– 2019845	MAD1L1	0	ALL	
39	ICR_439*^	chr7:5144439–5144757	chr7:5144438–5144493	ZNF890P	0	NHB	M
40	ICR_452^	chr7:45564015–45564354	chr7:45563920–45564036	ADCY1	9786	NHB	P
41	ICR_470^	chr7:73330191–73330782	chr7:73330658–73331092	FKBP6	0	NHB	
42	**ICR_473^**	**chr7:76150206**–**76150703**	chr7:76149513–76150704	GTF2IP7	41,444	NHB	
43	ICR_481*^# (A)	chr7:130490640–130494200	chr7: 130494195– 130494648	MEST|MESTIT1	0	NHB	M
	ICR_481*^# (B)	**chr7:130490640**–**130494200**	chr7: 130492063– 130492131	MEST|MESTIT1	0	NHW	M
	ICR_481*^# (C)	chr7:130490640–130494200	chr7: 130492246– 130492270	MEST|MESTIT1	0	ALL	M
	ICR_481*^# (D)	chr7:130490640–130494200	chr7: 130494195– 130494648	MEST|MESTIT1	0	ALL	M
44	ICR_484^	chr7:138664218–138664771	chr7:138664233–138664251	SVOPL	0	NHB	
45	ICR_491	chr7:155071148–155071376	chr7:155071182–155071231	HTR5A	0	NHB	M
46	ICR_533 (A)	chr8:57280020–57280373	chr8:57280257–57280374	LINC00588	0	NHB	P**
	ICR_533 (B)	chr8:57280020–57280373	chr8:57280257– 57280350	LINC00588	0	ALL	P**
47	ICR_545	chr8:110202766–110202966	chr8:110202860–110202920	RPSAP48	96,802	NHW	
48	ICR_548*^#	chr8:140098048–140100981	chr8:140098530–140098591	TRAPPC9|PEG13	0	NHB	M
49	**ICR_568**	**chr9:40584494**–**40584535**	chr9:40584526–40584688	AQP7P5	85,470	NHW	
50	**ICR_597**	**chr9:67720383**–**67720553**	chr9:67720318–67720505	FAM27E3	1204	NHB	
51	ICR_600	chr9:87944657–87944766	chr9:87944482–87944658	SPATA31C1	21,000	NHB	M
52	ICR_602	chr9:89859835–89860135	chr9:89860037–89860136	UNQ6494	140,076	ALL	P**
53	ICR_605 (A)	chr9:105519837–105519944	chr9:105519843–105519880	FSD1L|RALGAPA1P1	0	NHW	M**
	ICR_605 (B)	chr9:105519837–105519944	chr9:105519846– 105519890	FSD1L|RALGAPA1P1	0	ALL	M**
54	ICR_607^	chr9:110172756–110173053	chr9:110172902–110172965	PALM2-AKAP2	244	NHB|ALL	
55	**ICR_615^**	**chr9:127897741**–**127897833**	chr9:127897740–127898133	ST6GALNAC6	0	NHB	
56	ICR_621^	chr9:134800924–134801207	chr9:134800923–134801007	COL5A1	0	NHB	
57	**ICR_633^**	**chr10:5645451**–**5645631**	chr10:5645450–5645555	ASB13	0	NHB|ALL	P
58	**ICR_644^**	**chr10:28326170**–**28327001**	chr10:28326690–28326841	ZNF101P1	12,276	NHB	P
59	ICR_659	chr10:61867420–61867672	chr10:61867650–61867680	LINC02625	0	NHB	M**
60	ICR_664^	chr10:71266448–71266685	chr10:71266523–71267095	UNC5B	0	NHB	P
61	**ICR_684**	**chr10:125773898**–**125774247**	chr10:125774034–125774127	MMP21	0	NHW	
62	**ICR_710^**^**&**^	**chr11:400577**–**400771**	chr11:400612–400787	PKP3	0	ALL	
63	ICR_716*^#	chr11:1997886–1999417	chr11:1998628–1998714	MRPL23|H19	0	NHB	P
64	ICR_719*^#	chr11:2001655–2003118	chr11:2002634–2002682	MRPL23	0	NHW|ALL	P
65	ICR_744*^	chr11:96341424–96341836	chr11:96341176–96341443	MAML2|MIR1260B	0	NHB	
66	ICR_794^	chr12:110247427–110248050	chr12:110247783–110248328	IFT81	28,634	NHB	
67	**ICR_805^**	**chr12:130686671**–**130687021**	chr12:130685426–130687022	RIMBP2	0	NHB	
68	**ICR_808**	**chr12:132514078**–**132514147**	chr12:132513962–132514680	FBRSL1	0	NHB	P**
69	**ICR_814**^**&**^	**chr13:20142811**–**20142911**	chr13:20142817–20142897	GJA3	0	NHB	M
70	ICR_827*^	chr13:60267612–60268519	chr13:60267683–60267898	LINC00434	0	ALL	M
71	**ICR_829^**	**chr13:80654682**–**80655272**	chr13:80654681–80654939	PWWP2AP1	125	NHB	M
72	ICR_831^	chr13:100521893–100522472	chr13:100522193–100522667	PCCA	0	NHB	
73	ICR_832^ (A)	chr13:106490937–106491301	chr13:106491112–106491204	EFNB2	0	NHB	
	ICR_832^ (B)	chr13:106490937–106491301	chr13:106491112–106491536	EFNB2	0	ALL	
74	ICR_839*	chr13:113802775–113802965	chr13:113802908–113802957	TMEM255B	0	NHB	
75	ICR_843*	**chr13:114199984**–**114200221**	chr13:114200003–114200127	CFAP97D2	0	NHW	M**
76	ICR_873*^#	chr14:100824556–100828242	chr14:100827448–100827505	MEG3	0	ALL	
77	ICR_893*^^&^	chr15:24954592–24956828	chr15:24955432–24955502	SNHG1|SNRPN|SNURF	0	NHB	M
78	**ICR_902^**^**&**^** (A)**	**chr15:56007034**–**56007264**	chr15:56006890–56007288	CNOT6LP1	0	NHB	
	ICR_902^^&^ (B)	chr15:56007034–56007264	chr15:56007215– 56007288	CNOT6LP1	0	ALL	
79	ICR_914^	chr15:99476322–99476786	chr15:99476061–99476586	LINC02244	73,869	NHB	P
80	ICR_918 (A)	chr16:561123–561328	chr16:561079–563336	PRR35	0	NHB	
	**ICR_918 (B)**	**chr16:561123**–**561328**	chr16: 561079– 561329	PRR35	0	ALL	
81	ICR_922^	chr16:2038931–2039033	chr16:2038752–2038952	SLC9A3R2	0	ALL	P**
82	ICR_927*^ (A)	**chr16:3443280**–**3444094**	chr16:3443244–3443387	ZNF597|NAA60	0	NHW	
	ICR_927*^ (B)	**chr16:3443280**–**3444094**	chr16: 3443128– 3443337	ZNF597|NAA60	0	ALL	
83	ICR_935	chr16:31428164–31428239	chr16:31428174–31428193	COX6A2	0	NHB	M**
84	ICR_966*^	**chr16:67654637**–**67654865**	chr16:67654392–67654671	CARMIL2	0	NHB	
85	**ICR_976^**	**chr16:87517952**–**87518116**	chr16:87517903–87519115	ZCCHC14	24,928	NHB	P**
86	**ICR_978 (A)**	**chr16:88431177**–**88431330**	chr16:88431219–88433792	ZNF469	0	NHB	
	ICR_978 (B)	chr16:88431177–88431330	chr16:88431219– 88432676	ZNF469	0	ALL	
87	ICR_979^	chr17:335949–336142	chr17:335938–335966	RPH3AL	0	NHB|ALL	P**
88	**ICR_987**^**&**^** (A)**	**chr17:5771207**–**5771575**	chr17:5771278–5771364	NLRP1	186,698	NHB	M**
	**ICR_987& (B)**	**chr17:5771207**–**5771575**	chr17:5771284– 5771340	NLRP1	186,698	NHW	M**
	**ICR_987& (C)**	**chr17:5771207**–**5771575**	chr17:5771252– 5771364	NLRP1	186,698	ALL	M**
89	**ICR_1027 (A)**	**chr17:79517963**–**79518428**	chr17:79517720– 79517977	RBFOX3	0	NHB	P
	**ICR_1027 (B)**	**chr17:79517963**–**79518428**	chr17:79518326– 79518634	RBFOX3	0	NHB	P
	ICR_1027 (C)	chr17:79517963–79518428	chr17:79517916–79517973	RBFOX3	0	ALL	P
90	ICR_1045*^ (A)	chr18:79404293–79404545	chr18:79404213-79404841	NFATC1	0	NHB	P**
	ICR_1045*^ (B)	chr18:79404293–79404545	chr18:79404222 –79404294	NFATC1	0	ALL	P**
91	ICR_1046	chr18:79532208–79532443	chr18:79532261–79532411	NFATC1	2885	NHB	
92	ICR_1071^	chr19:3375239–3375520	chr19:3374971–3375300	NFIC	0	NHB	
93	ICR_1079	chr19:6509209–6509630	chr19:6509440–6509508	TUBB4A	6361	NHB	P
94	**ICR_1103^**^**&**^	**chr19:21568786**–**21569830**	chr19:21569406–21569812	ZNF429	27,629	NHW	
95	ICR_1104*^&^	chr19:21678108–21678334	chr19:21678184–21678290	MTDHP3	0	ALL	
96	ICR_1108^	chr19:30253854–30254259	chr19:30253405–30253912	ZNF536	0	NHB	
97	ICR_1139^(A)	chr19:55493632–55493692	chr19:55493661–55493693	SSC5D	0	NHB	
	ICR_1139^(B)	chr19:55493632–55493692	chr19: 55493661– 55493706	SSC5D	0	ALL	
98	ICR_1142*^#	**chr19:56837320**–**56841439**	chr19:56839300–56839440	ZIM2|PEG3|MIMT1	0	ALL	M
99	ICR_1187^&^	chr20:30892127–30892735	chr20:30892682–30892758	DUX4L38	1726	NHB	
100	ICR_1191*^	chr20:31547027–31548129	chr20:31547408–31547421	HM13|MCTS2P	0	ALL	M
101	**ICR_1192^# (A)**	**chr20:37520202**–**37521842**	chr20: 37520201– 37520271	BLCAP|NNAT	0	NHB	M
	**ICR_1192^# (B)**	**chr20:37520202**–**37521842**	chr20: 37520954 –37521054	BLCAP|NNAT	0	NHB	M
102	ICR_1206*^#	**chr20:58850158**–**58852357**	chr20:58851318–58851371	GNAS	0	NHB	M
103	ICR_1207*^ (A)	chr20:58853850–58856828	chr20: 58855056– 58855067	GNAS	0	NHB	M
	ICR_1207*^ (B)	**chr20:58853850**–**58856828**	chr20: 58854476– 58854553	GNAS	0	ALL	M
104	**ICR_1208^**	**chr20:58888275**–**58890270**	chr20:58890064–58890121	GNAS	0	NHW	
105	ICR_1217*	**chr20:63482662**–**63482947**	chr20:63482840–63483019	EEF1A2	5067	NHB	
106	ICR_1236^&^	chr21:8214822–8214873	chr21:8214757–8214857	RNA45SN2|RNA28SN2	0	NHW	
107	ICR_1241	chr21:8220792–8221021	chr21:8221013–8221077	RNA45SN2	1490	NHW	
108	ICR_1242	chr21:8226175–8226554	chr21:8226206–8226311	RNA45SN2	6873	NHW	
109	ICR_1244	chr21:8249017–8249144	chr21:8249128–8249371	RNA18SP5	4698	NHW	
110	ICR_1254	chr21:8259990–8260098	chr21:8259878–8260036	RNA18SP5	4286	NHW	
111	ICR_1266	chr21:8399142–8399444	chr21:8399429–8399518	RNA45SN3|RNA28SN3	0	NHW	
112	ICR_1269	chr21:8409174–8409553	chr21:8409173–8409454	RNA45SN3	6831	NHW	
113	ICR_1271	chr21:8432767–8432863	chr21:8432792–8432919	RNA45SN1	359	NHW	
114	ICR_1273	chr21:8434286–8434377	chr21:8434255–8434399	RNA45SN1	0	NHW	
115	ICR_1275	chr21:8437284–8437586	chr21:8436713–8437584	RNA45SN1|RNA18SN1	0	NHW	
116	ICR_1284	chr21:8453321–8453640	chr21:8453320–8453701	RNA45SN1	6749	NHW	
117	ICR_1377*^	chr22:42532792–42533280	chr22:42532982–42533635	RRP7A	12,996	NHB	P
118	**ICR_1389^**	**chr22:50482322**–**50482580**	chr22:50482321–50482623	ADM2	12,996	NHB	
119	ICR_1409*^	chrX:39813662–39814142	chrX:39813989–39814100	MIR1587	23,419	NHB	P**
120	ICR_1476^	chrX:153780931–153781167	chrX:153780908–153781001	SRPK3	0	ALL	P**

ICRs when the DMRs between AD cases and controls differed by ≥ 10% (Black) and ≥ 15% (Bold). In this subset of 45 ICRs, identified with a more stringent cutoff (≥ 15%), only one DMR (chr15:26670350-26670605) emerged that is not present with the 10% cutoff

^*^ICRs overlapping ENCODE annotated regions of CTCF binding [35]

^ ICRs overlapping ENCODE annotated regions of DNase I hypersensitivity [35]

^#^ICRs overlapping previously published ICRs of imprinted genes [35, 55–68]

^&^ICRs that overlap previously established regions of systemic interindividual variation (SIVs) [35, 91]

% Parental methylation denotes whether the paternal (P, sperm) or maternal (M, oocyte) allele of an ICR is methylated based upon both sperm and oocyte methylation data [35]

^**^Parental allele methylation of an ICR based upon only sperm methylation [35]

ε ICR_716 and ICR_719 overlap with the same known ICR proximity to *H19/MRPL23*; ICR_1206, ICR_1207 and ICR_1208 overlap with the same known ICR proximity to *GNAS*

**Fig. 2:**
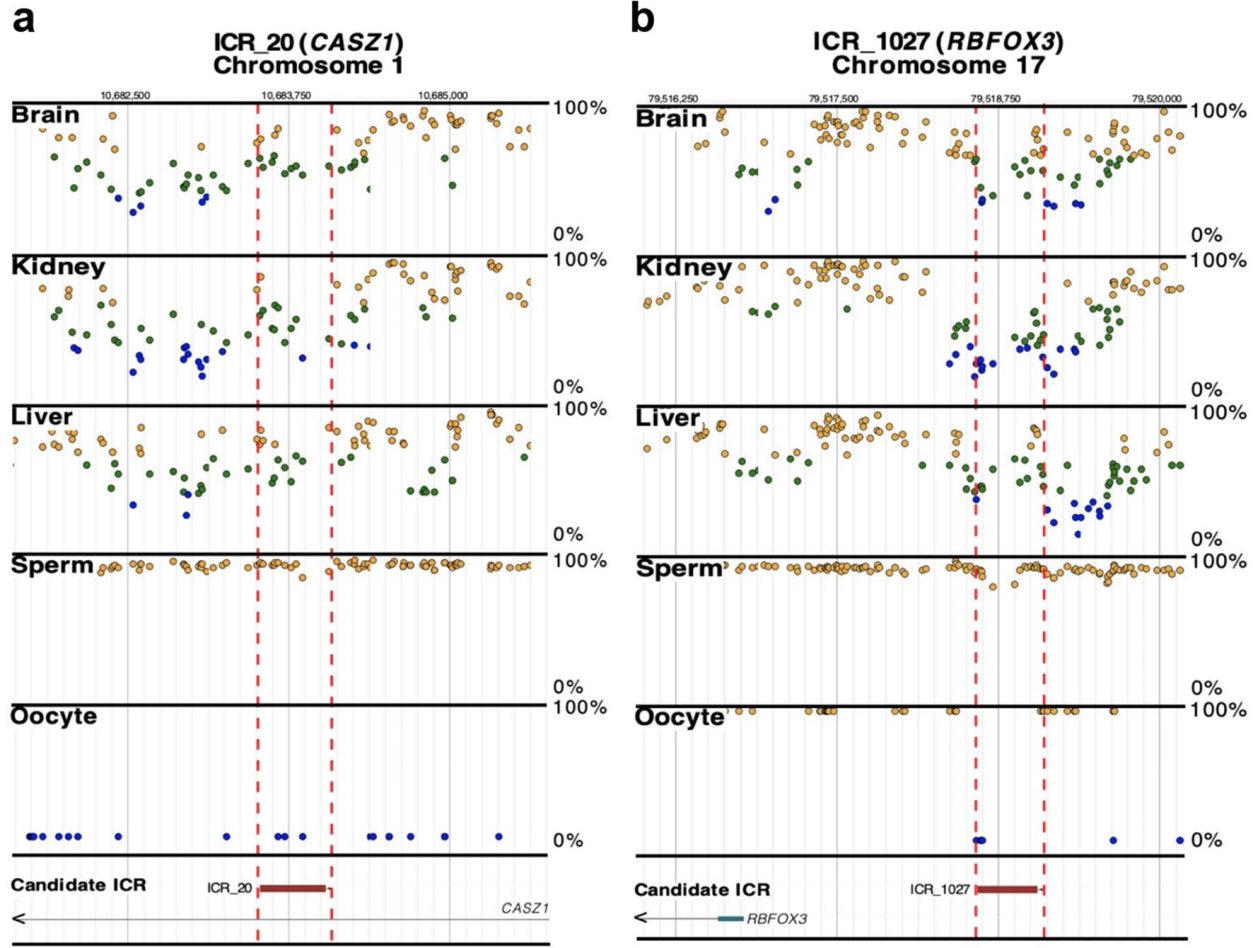
Race/ethnicity dependent ICRs in AD. **a** ICR_20 (*CASZ1*) and **b** ICR_1027 (*RBFOX3*) differed by ≥ 10% in DNA methylation between AD cases and controls only in NHBs. Candidate ICRs (horizontal red boxes) are delineated by vertical dashed red lines. The candidate ICRs were previously defined by having 5 or more consecutive CpGs with methylation levels of 50% ± 15% (green dots) for tissues in all three germ layers (*i.e.* brain, kidney, and liver); methylation levels for sperm and oocytes are also shown (*i.e.* ≥ 90% methylation—yellow dots and ≤ 10% methylation—blue dots) [35]

Notably, the AD-associated ICRs we identified are plausible targets for AD pathogenesis. For example, we found that ICR_20, near *CASZ1* (Fig. 2a) and ICR_1027, near *RBFOX3* (Fig. 2b) were differentially methylated in only NHBs. Methylation of DMRs overlapping ICR_20 (CDIF: 0.227, p-value: 1.07E-33) and ICR_1027 (CDIF: 0.208, p-value: 1.68E-25; CDIF: 0.166, p-value: 1.85E-16) were increased more than 10% in AD cases when compared to controls (Table 2). The *CASZ1* gene encodes the *Castor zinc finger 1* protein involved in neuronal differentiation [44]. An in vitro study demonstrated a gain of 5mC in the *CASZ1* region (chr1:10732049–10732050) in AD neurons compared to wildtype cells [45]. However, our results showed hypermethylation in AD brain samples compared to controls, in NHBs (chr1:10682586–10683160) and in ALL group (chr1:10682972–10683160) overlapping the *CASZ1* ICR (Table 1, Additional file 2: Table S2). *RBFOX3* encodes *NeuN* [46, 47], and is expressed in approximately 68% of cells in the gray matter of the cerebral cortex [47, 48]. Human brains affected by AD have decreased *RBFOX3* expression in the hippocampus when compared to non-AD brains [47]. Additionally, in a mouse study, *RBFOX3* was found to be developmentally regulated, and its expression is reported to coincide with *4R-tau* expression resulting from alternative splicing of tau exon 10 [49].

**Table 2:** AD associated differentially methylated regions overlapping ICR_20 (*CASZ1*), ICR_1027 (*RBFOX3*), ICR_987 (*NLRP1*) and ICR_481 (*MEST/MESTIT1*)

Gene ID	ICR_ID	DMR coordinates	Race/ethnicity	Mean methylation ratio in controls	Mean methylation ratio in ADs	Credible methylation difference (CDIF)	p−value	Methylation
CASZ1	ICR_20	chr1: 10682586–10683160	NHB	0.395	0.912	0.227	1.07E−33	Strong-Hyper
		chr1: 10682972–10683160	ALL	0.52	0.939	0.218	4.69E−23	Strong-Hyper
RBFOX3	ICR_1027	chr17: 79517720–79517977	NHB	0.319	0.869	0.208	1.68E−25	Strong-Hyper
		chr17:79518326–79518634	NHB	0.272	0.716	0.166	1.85E−16	Strong-Hyper
		chr17: 79517916–79517973	ALL	0.449	0.83	0.167	1.27E−09	Strong-Hyper
NLRP1	ICR_987	chr17:5771278–5771364	NHB	0.144	0.679	0.138	4.64E−21	Strong-Hyper
		chr17:5771284–5771340	NHW	0.162	0.828	0.218	1.26E−09	Strong-Hyper
		chr17:5771252–5771364	ALL	0.194	0.744	0.257	2.41E−50	Strong-Hyper
MEST|MESTIT1	ICR_481	chr7: 130494195–130494648	NHB	0.738	0.345	-0.159	1.08E−09	Strong-Hypo
		chr7: 130492063–130492131	NHW	0.69	0.178	-0.155	2.02E−14	Strong-Hypo
		chr7: 130492246–130492270	ALL	0.541	0.162	-0.137	2.16E−09	Strong-Hypo
		chr7: 130494195–130494648	ALL	0.73	0.443	-0.118	7.50E−09	Strong-Hypo

**Fig. 3:**
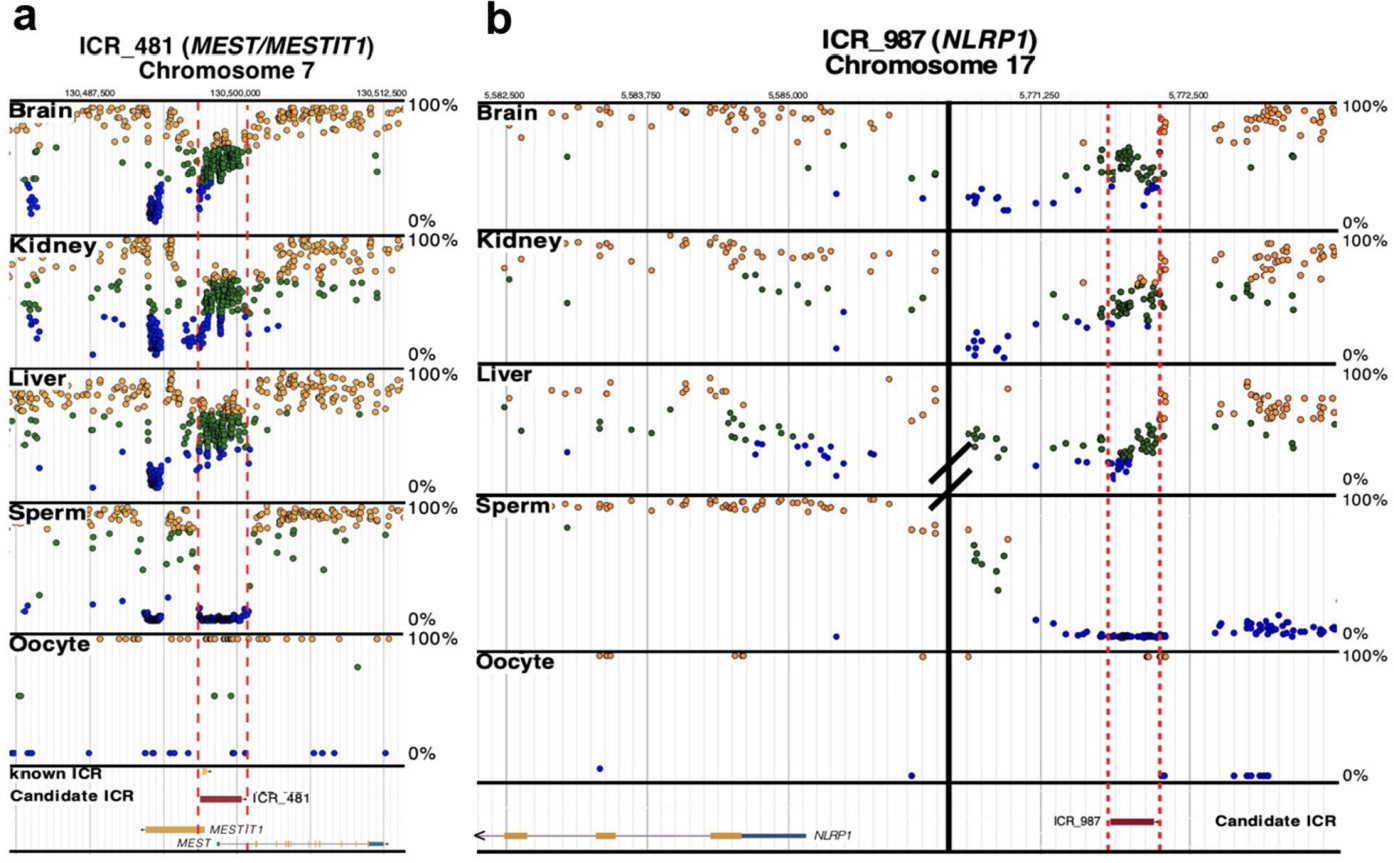
Race/ethnicity independent ICRs in AD. **a** ICR_481 (*MEST/MESTIT1*) and** b** ICR_987 (*NLRP1*) differed by ≥ 10% in DNA methylation between AD cases and controls in both NHBs and NHWs. Candidate ICR (horizontal red box) and a known ICR (horizontal yellow box) is delineated by vertical dashed red lines. The candidate ICR was previously defined by having 5 or more consecutive CpGs with methylation levels of 50% ± 15% (green dots) for tissues in all three germ layers (i.e., brain, kidney, and liver); methylation levels for sperm and oocytes are also shown (i.e., ≥ 90% methylation—yellow dots and ≤ 10% methylation—blue dots) [35]

Only two ICRs (ICR_481, chr7:130490640-130494200 and ICR_987, chr17:5771207-5771575), proximal to *MEST/MESTIT1* and *NLRP1,* respectively, were differentially methylated in both NHBs and NHWs (Fig. 1b and Fig. 3a, b). Methylation of ICR_481 (CDIFs: -0.159, -0.155, -0.137, and -0.118, p-values: 1.08E-09, 2.02E-14, 2.16E-09, and 7.50E-09) is decreased, whereas methylation of ICR_987 (CDIFs: 0.138, 0.218, and 0.257, p-values: 4.64E-21, 1.26E-09, and 2.41E-50) is increased more than 10% in AD cases when compared to controls (Table 2). *MEST* is a paternally expressed imprinted gene and highly expressed in mesoderm and adult brain [50]. It is linked to intrauterine growth retardation and abnormal maternal behavior in adult mice [51]. In humans, the maternal uniparental disomy related to imprinting at the *PEG1/MEST* region located at 7q32 causes Silver-Russell syndrome [52]. The *NLR family pyrin domain-containing 1* (*NLRP1*) inflammasome is widely expressed in humans [53]. In the central nervous system, it is primarily expressed by pyramidal neurons and oligodendrocytes where overexpression triggers caspase 1 and 6 activation, eventually leading to axonal degeneration and neuronal death by pyroptosis, an inflammatory form of programmed cell death [53, 54]. Repeating these analyses using a more stringent cutoff of 15% for a methylation difference reduced the total number of AD-associated ICRs from 120 to 45. Furthermore, it is noteworthy that ICR_987 maintained its differential methylation status in both NHBs and NHWs under the more stringent conditions. On the other hand, with a 15% cutoff ICR_481 was differentially methylated in only NHWs (Fig. 1c and Table 1).

When we constrained the analysis of the 1488 ICRs, specifically to the 332 for which gametic methylation patterns were available [35], we did not observe differences that would affect the proportional differences between AD and controls in both NHBs and NHWs. For example, we identified 37 of 332 candidate ICRs associated with AD, with 31 ICRs found in NHBs and two ICRs found in NHWs (Additional file 1: Table S3). Notably, we again observed a common ICR, ICR_481 (*MEST/MESTIT1*), which exhibited differential methylation in AD brains compared to controls. Despite observing a reduced number of DMRs associated with AD when constraining the analysis to the 332 ICRs for which there is gametic data, the demonstration of parental origin of methylation strengthens our confidence in the 37 identified differentially methylated regions as robust candidates for regulatory regions in the development and progression of AD.

We identified 106 genes in closest proximity to the 120 AD-associated ICRs (i.e., between 0 and 186,698 bp; average 11,270 bp) (Table 1). This is well within the range of known imprinted domains. For example, the *H19/IGF2* and *KCNQ1* imprinted domain is about 1.4 Mb long; that of *MEST/MESTIT1* is around 4.0 Mb; and that of the *NPP5F_v2* is nearly 8.6 Mb DNA [55].

When stratified by race/ethnicity, there were 85 NHB-AD associated ICRs and 26 were linked to NHW-AD associated ICRs. Network analysis conducted separately on the 85 ICRs in NHBs and 26 ICR in NHWs using ingenuity pathway analysis (IPA) unveiled shared functions such as cell signaling, cellular development, embryonic development, and organ development in both NHBs and NHWs (Additional file 1: Table S4, S5). Interestingly, two pathways, namely white adipose tissue browning and gap junction signaling, were also identified as common features in both NHBs and NHWs. On the other hand, the netrin signaling pathway, known to regulate axonal growth, was found only in NHBs.

The 106 genes linked to the 120 AD-associated candidate ICRs contained 16 previously known imprinted genes [56] (http://www.geneimprint.com) in close proximity to 13 AD-associated candidate ICRs. Of these imprinted genes, nine (*MEST* [57, 58]*, MESTIT1* [58], *PEG13* [59]*, SNRPN* [60]*, SNURF* [61]*, ZIM2* [62, 63]*, PEG3* [62, 63]*, MIMT1* [63]*, NNAT* [64]) are paternally expressed, five (*SVOPL* [65]*, H19* [66]*, MEG3* [67], *ZNF597* [68], *NAA60* [68]) are maternally expressed, and two (i.e., *BLCAP* [69]*, GNAS* [70]) have isoform-dependent expression (71). This indicates that monoallelic parent-of-origin expression can present in some gene transcripts (i.e., isoforms), but not in others.

### lncRNAs and microRNAs analyses

Many known epigenetic regulators of gene expression include lncRNAs and microRNAs (miRNAs). Thus, we examined the lncRNAs associated with the 120 ICRs linked to AD using LncExpDB [72]. We found 9 lncRNAs (*BABAM2-AS1, LINC01101, HTR5A-AS1, PEG13, FAM27E3, H19, PCCA-DT, SNHG1, and GNAS-AS1*) within or near differentially methylated AD-associated ICRs in NHBs and only one lncRNA (*GNAS-AS1*) in NHWs, all of which are linked to brain development. To identify microRNAs overlapping the 120 ICRs, we used *hsa.gff3* data provided by miRbase [73], and found two microRNAs (i.e., *miR1260b, miR1587*) in NHBs among the annotated genes. *miR1260b* is reported to regulate two tumor suppressor genes, *sFRP1* and *SMAD4*, in prostate cancer through epigenetic mechanisms [74, 75], and to also extensively participate in arthritis, osteogenic differentiation, and Alzheimer's disease [76].

## Discussion

Although it is established that mutations in *APOE, APP, PSEN1/2,* and *BACE1* contribute to AD risk [77, 78], and that the *APOE-ε4* allele affects cognitive function [77, 79], known genetic variation alone explains only a small proportion of AD. Evidence in the last decade supports that epigenetics may contribute substantially to altered gene function and disease development. The highly conserved and stable methylation pattern of ICRs makes them particularly valuable in the study of diseases like AD, which do not manifest until adulthood or advanced age. After fertilization and remodeling of methylation status during the intrauterine period, ICRs normally maintain the same methylation status in all cells and tissues, including in blood and brain tissue, throughout life. For this reason, early changes in the methylation of ICRs could potentially serve as susceptibility biomarkers for disease risk, and they could be measured at any time during an individual's life. To elucidate the AD associated candidate ICRs in brain tissue that have potential regulatory functions, we determined the methylation pattern of the genome by WGBS and identified the differentially methylated regions in AD brain samples compared to that in controls. We identified 120 candidate ICRs with altered methylation levels in patients with AD.

We next determined whether the patterns of AD-related methylation in candidate ICRs differ by racial/ethnic group. A threefold difference in the number of AD-related ICRs was found in the brain samples of NHBs (67.5%) when compared to NHWs (22.5%). This finding is consistent with the postulate that environmentally responsive epigenetic differences in the methylation of ICRs could contribute to the racial/ethnic disparities observed in AD between NHBs and NHWs [1].

Remarkably, one of the two common ICRs identified in NHBs and NHWs was ICR_481 (*MEST/MESTIT1*). *MEST/PEG1*, a paternally expressed imprinted gene, was shown to regulate neuronal migration in development of neocortex [80, 81], block neuron differentiation when knocked out, and inhibit Wnt signaling when expressed [50]. This is functionally significant, as Wnt signalling has been reported to be associated with age related neurodegenerative diseases including AD [80, 82]. As *MEST* expression is reduced by promoter hypermethylation, this would result in activation of Wnt signaling in brain tissues of AD patients, potentially facilitating the progression of AD [80].

The second ICR identified in NHBs and NHWs was ICR_987. It is closest to the gene *NLRP1,* a component of the inflammasome complex that triggers an immunostimulatory form of cell death called pyroptosis, which is activated in neuronal cells in response to amyloid-β (Aβ) aggregates [53, 83]. Inflammasomes are multiprotein complexes that are assembled in response to a cellular stressor, including infection, and lead to caspase activation [84]. They also are associated with neurodegenerative diseases such as AD [53], consistent with the hypothesis that neuroinflammation contributes substantially to neurodegeneration [53]. Studies in murine AD models indicate that the *Nlrp1* inflammasome is indeed upregulated, and neuronal death is observed, leading to cognitive decline [53]. Kaushal et al. [54] reported a 25- to 30-fold higher number of *NLRP1*-expressing neurons in AD brains compared to control brains. The existence of significantly increased methylation of the candidate ICR_987 (*NLRP1*) in both NHBs and NHWs supports the neuroinflammation hypothesis of AD formation, one of the most studied mechanisms in AD pathogenesis.

Furthermore, there are four single nucleotide polymorphisms (SNPs) (rs2137722, rs11657747, rs34733791 and rs11651595) in the *NLRP1* region that have been reported as significantly associated with AD [85]. The major alleles for SNPs rs2137722 and rs11657747 are Gs in CpG sites, such that the minor alleles abolish potential methylation sites. Interestingly, the minor A allele for rs2137722, which would block methylation, appears to provide a protective effect against AD [85]. This functional importance of *NLRP1* in the development of AD further supports the potential regulatory importance of the proximal differentially methylated ICR_987.

We conducted a comparative analysis of data from three previous epigenome studies: Zhang et al. (2020) in prefrontal cortex [86], Smith et al. (2021) in prefrontal cortex, temporal gyrus, and entorhinal cortex [87], and Breen et al. (2023) in blood [88]. We aimed to identify overlaps between previously reported associations and DMRs identified in this study (Additional file 2: Table S2). The first two studies (Smith et al. (2021) [87] and Zhang et al. (2020) [86]) utilized methylation data from the Illumina HumanMethylation 450 k beadchip, from which we identified commonalities with our AD-associated DMRs (Additional file 3: Table S6). However, none of the 120 candidate ICRs overlapped the differentially methylated positions (DMPs) reported in these two studies. A limiting factor in using the Illumina HumanMethylation 450 k beadchip array is that coverage is for approximately 450,000 methylation sites, constituting only 3% of the total 28,084,558 CpGs in the human genome. Comparison of the 450 K manifest with the coordinates of the AD-ICRs identified 102 CpG sites in common, in 45 ICRs. The lack of common regions between these different approaches is at least partially attributable to this low coverage.

The study by Breen et al*.* (2023) employed whole-genome methyl-sequencing in blood and looked at differentially methylated positions (DMPs) in AD patients compared to controls [88]. The comparisons between the DMRs identified in this recent study and our AD associated DMRs listed in Table 1 revealed 102 overlapping DMRs in NHBs, including DMR chr7:76149513–76150704, which overlaps with candidate ICR_473, two DMRs in NHWs, and 22 DMRs in ALL group that are common in both studies (Additional file 3: Table S6).

Additionally, an epigenome-wide association study (EWAS) conducted by Piras et al*.* [89] revealed differential methylation patterns in the genes of AD brains as compared to non-demented control brains. The study identified 832 DMRs, out of which five DMRs were found associated with *CASZ1, MAD1L1, MRPL23, RIMBP2, SLC9A3R2, ZCCHC14,* and *NFIC*. Interestingly, these genes were each in proximity to an ICR identified in our study (ICR_20, ICR_434, ICR_716/ICR_719, ICR_805, ICR_922, ICR_976, and ICR_1071, respectively; see Table 1). There are other epigenome-wide studies that showed DMRs associated with various regions of the brain [87, 90]. However, more studies with a focus on ICRs and higher coverage are needed to understand the association between ICRs and AD development.

The AD-associated ICRs described here also overlap other known early-established methylation-dependent gene regulatory regions. A previous study determined regions of systemic interindividual variations (SIVs), characterized by consistent methylation across tissues within individuals, but with significant variation among individuals [91]. Like ICRs, SIV methylation is established before tissue specification, hence the consistency within individuals, but unlike ICRs, methylation is not restricted to parent-of-origin status. The SIV control regions comprise approximately 0.1% of the human genome and regulate the expression of metastable epialleles. The most widely known example of a metastable epiallele is the agouti viable yellow (A^vy^) locus in mice used to demonstrate that environmentally induced epigenetic modifications during early development can produce a range of phenotypes and alter disease susceptibility in adulthood [24]. SIVs are defined as regions “conserved across diverse human ethnic groups, sensitive to periconceptional environmental exposures, and associated with genes implicated in a broad range of human disorders and phenotypes” [91, 92]. Interestingly, 15 of the AD-associated candidate ICRs overlap with previously described SIVs (Table 1) [91], including ICR_987 (chr17:5771207–5771575, *NLRP1*), common to both NHB-AD and NHW-AD case control comparisons.

Obesity is known as one of the modifiable risk factors for dementia [93]. According to a study by Nianogo et al. [2] one third of the AD related dementia cases were linked to a combination of modifiable risk factors, including midlife obesity, physical inactivity, and educational attainment [2]. In support of this, our analysis revealed the white adipose tissue browning pathway as a common enriched pathway associated with AD for both NHB and NHW populations. Changes in adipose tissue are part of the normal aging process [94], as adipose tissue is highly dynamic and has a role in homeostatic processes. White adipose tissue (WAT) may actively change into beige or brown adipose tissue (BAT) with environmental factors [95] through the white adipose tissue browning pathway. WAT is known to function as an endocrine organ secreting various types of adipokines including *TNFα*, which increases with aging. High WAT mass is related to metabolic disorders and linked to insulin resistance [94], and high BMI is associated with a reduction in brain volume [96, 97]. It is suggested that impairment in adipose tissue-derived adipokines may cause problems in brain homeostasis [94], which may eventually lead to neurodegenerative diseases.

Additionally, we identified the gap junction signaling pathway in both NHB and NHW populations as an enriched pathway, which is potentially dysregulated in AD. *GNAS*, an imprinted gene that is involved in multiple signaling pathways associated with G protein-coupled receptors, is also one of the molecules involved in the gap junction signaling pathway. Transcription of connexins, involved in the gap junction signaling pathway, are reported to be regulated by epigenetic modifications [98]. *GJA3*, in close proximity to ICR_814 (chr13:20142811–20142911), is a member of the gap junction signaling pathway and was found to contain a differentially methylated CpG (chr13: 20736075) in AD hippocampus samples compared to controls [99]. Future studies on the possible epigenetic regulation of these pathways may elucidate their mechanisms in the development of AD.

Interestingly, our network analysis identified netrin signaling in NHBs only. Netrins are axon guidance molecules associated with the regulation of axonal growth and play roles in neuroinflammation. *Netrin 1* is reported to inhibit Aβ production [100]. *UNC5B*, a molecule belonging to the netrin signaling pathway, was the closest gene to candidate ICR_664 (chr10:71266448–71266685), which was hypomethylated in AD brain samples compared to controls (Table 1, Additional file 2: Table S2). The *UNC5B* receptor is activated by the *Netrin 1* molecule in the netrin signaling pathway. It is reported to have inhibitory roles in the inflammatory response in nervous system and may have a protective role in neurodegeneration [100, 101].

A major strength of our study is that it is one the largest investigations, with deeply phenotyped participants with WGBS data, that uses DNA derived from brain samples not only from NHWs, but also NHBs, who are rarely included in the study cohorts, despite their higher risk of developing AD. Nevertheless, the data should be interpreted in the context of its limitations. Firstly, the relatively small sample size (n = 17) could diminish the statistical power required to identify some ICRs associated with AD. Despite the sample size, our use of agnostic WGBS revealed novel changes in ICRs that should be detectable in accessible tissues. Moreover, WGBS has revealed racial/ethnicity-dependent differences in ICR methylation that may lead to a better understanding of disparities in AD. Further analysis of this phenomenon may improve the treatment of AD by providing a mechanism for determining at-risk individuals. While the inclusive nature of our study aimed to encompass a diverse cohort, the small sample size also introduces an inevitable limitation, preventing us from drawing definitive conclusions regarding sex-specific ICR methylation patterns in the context of AD. However, further analyses with a larger sample size to address this issue in future studies are necessary.

Secondly, brain structural changes in AD start in the entorhinal cortex and medial temporal lobes and extend into the neocortex [4] and cerebellum [102] over time. We cannot exclude the possibility that the threefold difference in the number of differentially methylated ICRs associated with AD is due, in part, to epigenetic differences given the heterogeneity in NHB control tissues, which were a combination of temporal cortex and cerebellum. However, ICR methylation should be similar across tissue and cell types and should not be affected by tissue/cell heterogeneity. For that reason, we are confident with our definition of differentially methylation in candidate ICRs.

We also repeated our initial analyses using the more stringent DMR cutoff of 15% to reduce false positives and restricted our analysis to a subset of the 1488 ICRs previously reported, specifically the 332 ICRs with gametic methylation data. This more stringent analysis did not alter the findings that aberrant ICRs methylation is over-represented among AD cases, more so in NHBs than NHWs. Although differential ICR methylation holds promise in surveillance to identify AD, replicating these findings in high-powered studies with DNA derived from various brain regions and accessible tissues such as blood or central nervous system fluids, in diverse populations, are necessary.

Finally, while we acknowledge the existence of epigenetic drift associated with aging, we lack information regarding the methylation status of ICRs throughout the aging process. ICRs represent specific regulatory regions, and the DNA methylation patterns established during the intrauterine stage remain conserved. Furthermore, a study conducted by Mancino et al. (2023) on the hippocampus of mice, highlights a notable age-related increase in DNA methylation—an established transcriptional indicator of aging [103]. Interestingly, the authors observed that genomic imprinting, specifically parent-of-origin-specific DNA methylation, remained largely unaffected by the aging process. This observed stability extended across various brain regions, including the cerebellum, nucleus accumbens, hypothalamus, and prefrontal cortex. Transcriptomic analysis further substantiated these findings, confirming the preservation of imprinted expression in the aged hippocampus [103]. Nevertheless, our current dataset does not provide insights into age-related changes. With the anticipation of acquiring more comprehensive data through larger sample sizes and long-term follow-up studies, we aim to unravel the methylation dynamics of ICRs in the future, drawing from an extended population.

## Conclusion

Using unbiased WGBS, we provide the first evidence that DNA methylation in 120 ICRs varies markedly between AD cases and controls. The number of ICRs with altered methylation is three times higher in NHBs with AD than in NHWs with AD, which may contribute to the higher prevalence of AD in NHBs compared to NHWs [1]. Our findings are also consistent with the *developmental origins of health and disease* (*DOHaD*) hypothesis that increased susceptibility to adult-onset chronic diseases such as AD frequently have their origins in early development [104], and support the findings that AD is characterized by changes in the brain that likely start decades before the clinical symptoms appear [40, 41]. Thus, alteration in ICR methylation may serve as an early detection tool of AD risk that is essential for slowing the progression of this disease.

## Methods

### Human brain specimens

To identify differentially methylated ICRs associated with AD, we obtained frozen autopsy brain specimens from the Joseph and Kathleen Bryan Brain Bank of the Duke University/University of North Carolina at Chapel Hill Alzheimer’s Disease Research Center (Duke/UNC ADRC); informed consent documentation is in IRB ID# 00016278. The AD and control brain tissues were selected according to their neuropathologic diagnosis of AD. Nine brain samples from AD cadavers (five NHBs and four NHWs) and eight brain samples from control cadavers (four NHBs and four NHWs) were collected. For all individuals, the time elapsed between patient death and collection and snap-freezing of samples was less than 24 h (Additional file 1: Table S1).

### Library preparation of specimens, WGBS, and identification of AD-associated DMRs

We performed WGBS to identify DMRs associated with AD in all brain samples (n = 17). Libraries were prepared from extracted DNA using EpiGnome™ Methyl-Seq reagents (Illumina, Inc, *San Diego, CA*), index-tagged for multiplexing, and sequenced on an Illumina NextSeq platform (Illumina, Inc, *San Diego, CA*). Reads were assigned back to individuals by index reads, and aligned in silico to a bisulfite-converted reference genome (i.e., hg38 version 87). Reads without unique alignments, due to either repetitive genomic sequence or loss of specificity from bisulfite conversion of cytosines, and duplicate reads, indicative of clonal amplification of original random DNA fragments, were eliminated. The quality of each paired-end sequence file was inspected using *fastqc*, and adapter trimming and quality control were performed using the *Trim Galore* wrapper script that calls the *cutadapt* [105] script internally. Paired-end reads with a trimmed adapter sequence were aligned to the human reference genome (i.e., hg38 version 87) downloaded from Ensembl using *bsmap aligner* [106]. The following options were passed to the aligner: *-p 8 -L 135 -w 100 -v 10 -q 10 -R -V 1.* The aligned bam files were sorted and indexed using *samtools* [107], and duplicate sequence reads were removed using the *Picard* [108] application.

**Fig. 4:**
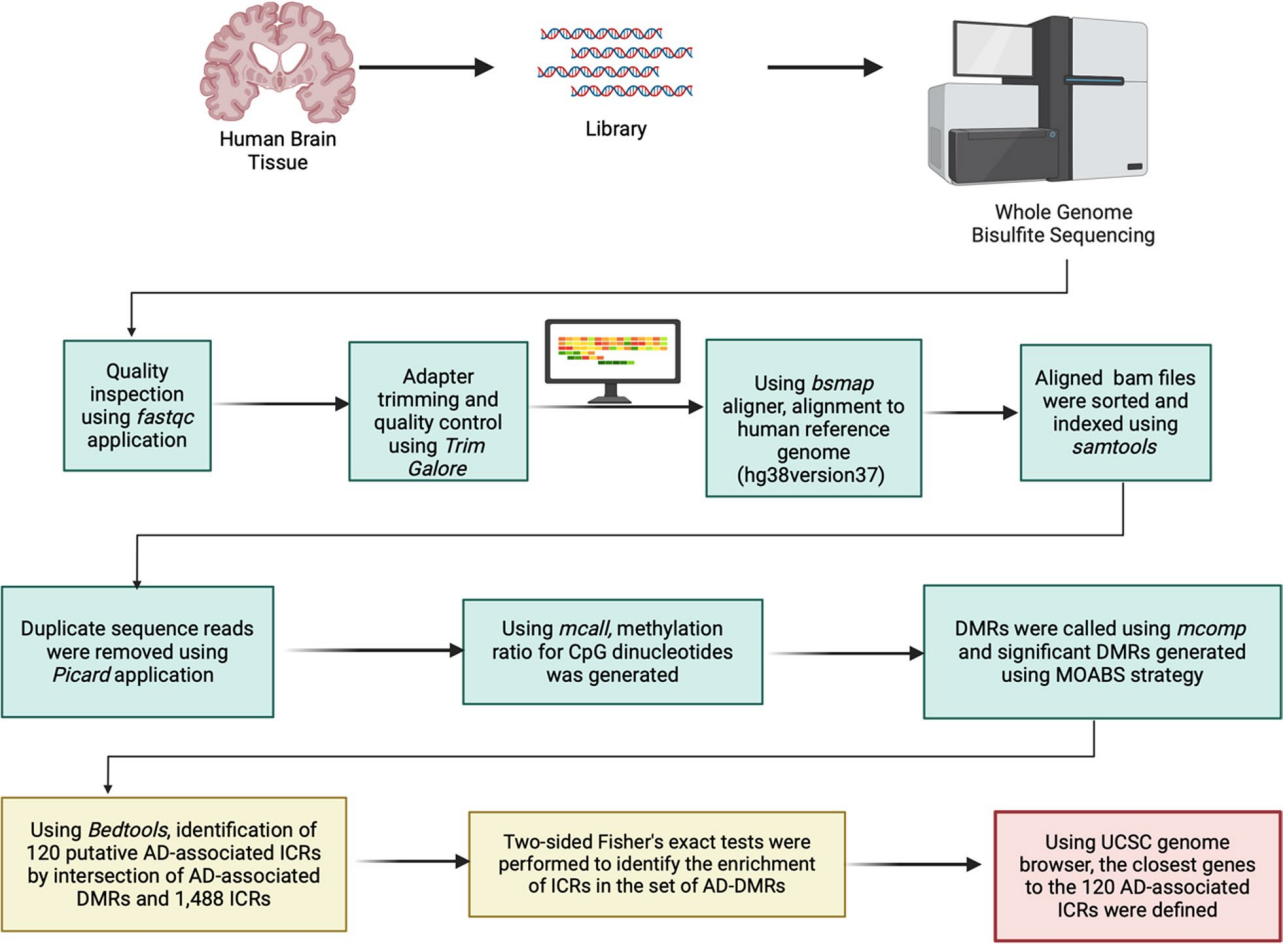
Experimental workflow. The steps used to identify DMRs in AD cases vs controls, overlapping ICRs, and closest genes. Created with BioRender.com

We analyzed three groups from 17 study participants whose clinic and demographic characteristics are summarized in Additional file 1: Table S1. These included: I) All AD samples vs. all controls, II) NHB-AD vs. NHB-control samples, III) NHW-AD vs. NHW-control samples (Fig. 1a). Briefly, a methylation ratio for CpG dinucleotides was generated from replicate bam files for each AD samples and controls using *mcall,* and DMRs were called between AD samples and controls using *mcomp*. The following options were utilized for *mcomp* inputs: *–doStrandSpecifiMeth* = *1, –doDmrScan* = *1, –doDmcScan* = *1, –dmrMethods* = *2 –minDmcsInDmr* = *4, –minCredibleDif* = *0.1 or 0.15, –maxDistConsDmcs* = *300, –minDepthForComp* = *7 –pFetDmc* = *0.05.* DMRs were called using Model-based analysis of bisulfite sequencing data (MOABS, version 1.3.8.7), which relies on Credible Methylation Difference (CDIF) as a single metric for both statistics and biological significance of differential methylation, i.e., significant DMRs were generated using MOABS, and confirmed with known DMRs as positive controls [42]. The following criteria were used in calling a DMR: minimum differentially methylated C’s $\ge$ 4, credible *cis*-acting methylation difference $\ge$ 10% or $\ge$ 15%, minimum read depth $\ge$ 7, and max distance between consecutive CpG’s $\le$ 300 (Fig. 4). After merging the bam files, the total coverage from certain CpGs divided by total coverage for all CpG’s (wig sum percentages) were plotted versus increasing read depth using *mmint* (https://github.com/lijiacd985/Mmint) (Additional file 1: Fig. S1a). This plot helped us check if there were high duplication levels or sequence bias. There was no sequence bias observed for all AD and control samples. We have generated the coverage plot using the *plot coverage* function from *deepTools* [109] using the merged bam files per group (Additional file 1: Fig. S1b, c).

### Identification of AD-related ICRs in NHBs and NHWs

The DMRs associated with AD were intersected with the recently defined ICRs [35] using *bedtools*. A description of the process for defining human ICRs was recently published [35]. Briefly, *puticr,* a custom tool implemented in Python (Version $\ge$ 2.7), was used to identify 1488 ICRs in tissue from multiple germ layers [35]. We used *Bedtools* [110] to identify ICRs that intersected with AD-associated DMRs, and then used two-sided Fisher’s exact test to identity enrichment of ICRs in the set of AD DMRs (Fig. 4).

To explore the functional significance of these AD-associated ICRs, genes closest to either side of the 120 AD-associated ICRs were identified from the UCSC genome browser. To explore molecular and cellular functions, potentially enriched pathways, and associations with disease, we analyzed our gene set using online tools, including *Ingenuity Pathway Analysis (IPA)* [111].

*Microsoft Excel v16.46* (21021202) and *Bedtools* v2.30.0 were used to compare ICR coordinates with published data. Using the *Expression Database of Human Long noncoding RNAs (LncExpDB)*[72], we compared lncRNAs associated with brain development genes, and the genes in close proximity to ICRs. Furthermore, using *hsa.gff3* data provided by *miRbase* [73], we identified microRNAs among the annotated genes that overlap with AD-associated ICRs.

### Supplementary Information

**Additional file 1: Table S1.** This table provides demographic and clinical characteristics of both Alzheimer's disease (AD) cases and controls from which the brain samples were obtained. Table S3. This table presents a compilation of AD-associated differentially methylated regions (DMRs) that overlap with 332 candidate inherited ICRs, which have been validated through parental allele confirmation. Table S4 and S5. These tables contain results from the functional and pathway analysis of genes that are in close proximity to AD-associated ICRs. Table S4 pertains to non-Hispanic Black (NHB) populations, while Table S5 focuses on non-Hispanic White (NHW) populations. Figure S1. This figure shows the results of the quality control for the WGBS data, ensuring the reliability and accuracy of the obtained results.

**Additional file 2: Table S2.** The supplementary materials comprise additional details of the DMRs identified across three distinct comparison groups, the overall group (ALL), NHBs, and NHWs, including: Chromosome number: Indicating the chromosome on which the DMR is located. DMR coordinates: Providing the precise coordinates of the DMR on the chromosome. Mean ratio: The average methylation ratio across the DMR region. Cytosine number: Specifying the count of cytosines within the DMR. Number of CpG sites: Enumerating the quantity of CpG sites present within the DMR for both controls and AD patients. Methylation difference: Representing the difference in methylation levels between AD patients and controls. p-value: the statistical significance of the observed methylation differences. Hyper/hypomethylation status: Indicating whether the region is hypermethylated or hypomethylated in AD patients relative to controls.

**Additional file 3: Table S6.** The supplementary materials comprise additional details of the DMRs shared between AD-associated DMRs identified in our study in brain stratified by race/ethnicity and the study by Breen et al. (2023) in blood [88].

## Data Availability

The raw sequencing data obtained by our study are in the process of being submitted to dbGaP.
