# Supplementary material for: DNA methylation of imprint control regions associated with Alzheimer’s disease in non-Hispanic Blacks and non-Hispanic Whites
Source: Clin Epigenetics. 2024 Apr 25;16:58. doi: 10.1186/s13148-024-01672-4 (PMC11043040; doi:10.1186/s13148-024-01672-4)
Supplement: Supplementary file 1 — Additional file 1: Table S1. This table provides demographic and clinical characteristics of both Alzheimer's disease (AD) cases and controls from which the brain samples were obtained. Table S3. This table presents a compilation of AD-associated differentially methylated regions (DMRs) that overlap with 332 candidate inherited ICRs, which have been validated through parental allele confirmation. Table S4 and S5. These tables contain results from the functional and pathway analysis of genes that are in close proximity to AD-associated ICRs. Table S4 pertains to non-Hispanic Black (NHB) populations, while Table S5 focuses on non-Hispanic White (NHW) populations. Figure S1. This figure shows the results of the quality control for the WGBS data, ensuring the reliability and accuracy of the obtained results. [file 13148_2024_1672_MOESM1_ESM.docx]

**Supplementary Table S1. Demographic and clinical characteristics of AD cases and controls from whom brain samples were derived.**

| **Sample ID** | **AD Case/Control** | **Ethnicity** | **Age** | **Sex** | **Brain Region** | ****APOE* Variants** | **AD Neuropathologic Changes** | **Time from Death to Freezing**  **(hour/minute)** |
| --- | --- | --- | --- | --- | --- | --- | --- | --- |
| 312 | AD Case | NHB | 84 | Male | Temporal Cortex | E33 | High | 05:13 |
| 411 | AD Case | NHB | >89 | Female | Temporal Cortex | E34 | Intermediate | - |
| 760 | AD Case | NHB | 77 | Female | Temporal Cortex | E44 | Intermediate | 04:00 |
| 1381 | AD Case | NHB | 88 | Female | Temporal Cortex | E34 | High | 04:15 |
| 1620 | AD Case | NHB | 63 | Female | Temporal Cortex | E34 | High | 04:05 |
| 1594 | AD Case | NHW | 88 | Male | Temporal Cortex | E33 | Intermediate | 03:55 |
| 1665 | AD Case | NHW | 86 | Female | Temporal Cortex | E34 | AD | 08:15 |
| 1696 | AD Case | NHW | >89 | Male | Temporal Cortex | E34 | Intermediate | 04:40 |
| 1705 | AD Case | NHW | 77 | Female | Temporal Cortex | E33 | AD | 18:15 |
| 94 | Control | NHB | 67 | Female | Cerebellum | E23 | None | 00:40 |
| 158 | Control | NHB | 56 | Male | Cerebellum | E24 | None | 19:27 |
| 328 | Control | NHB | 69 | Male | Cerebellum | E33 | None | 01:05 |
| 591 | Control | NHB | 78 | Female | Temporal Cortex | E33 | None | 05:31 |
| 1545 | Control | NHW | 64 | Female | Temporal Cortex | E33 | None | 10:40 |
| 1600 | Control | NHW | 85 | Female | Temporal Cortex | E33 | None | 12:58 |
| 1609 | Control | NHW | 86 | Male | Temporal Cortex | E33 | Low | 16:20 |
| 1678 | Control | NHW | 87 | Male | Temporal Cortex | E23 | None | 17:48 |

**APOE* Variants E2, E3, E4

NHB - Non-Hispanic Black

NHW - Non-Hispanic White

**Supplementary Table S3. AD-associated DMRs overlapping with 332 candidate ICRs confirmed with parental allele.**

| **Number** | **ICR** | **ICR Coordinates** | **DMR Coordinates** | **Nearest Transcript** | **Distance to closest gene (bp)** | **Race**  **Ethnicity** | **^%^Parental Methylation** |
| --- | --- | --- | --- | --- | --- | --- | --- |
| 1 | ICR_17^ | chr1:8117511-8117827 | chr1: 8117511 -8117532 | RPL7AP18 | 59024 | NHB | P |
| 2 | ICR_20^ (A) | chr1:10682902-10683413 | chr1: 10682586- 10683160 | CASZ1 | 0 | NHB | P |
|  | ICR_20^ (B) | chr1:10682902-10683413 | chr1: 10682972- 10683160 | CASZ1 | 0 | ALL | P |
| 3 | ICR_39 | chr1:38210131-38210429 | chr1:38210102-38210802 | LINC01343 | 0 | NHB | P |
| 4 | ICR_116^ | chr2:28342638-28342806 | chr2:28342650-28342769 | BABAM2 | 3737 | NHB | P |
| 5 | ICR_125 (A) | chr2:54289850-54290281 | chr2: 54289947 -54290188 | ACYP2 | 0 | NHB | M |
|  | ICR_125 (B) | chr2:54289850-54290281 | chr2: 54289947- 54290255 | ACYP2 | 0 | ALL | M |
| 6 | ICR_144 | chr2:120526146-120526533 | chr2:120525904-120526415 | LINC01101 | 59797 | NHB | P |
| 7 | ICR_188 | chr2:241902453-241902725 | chr2:241902629-241902778 | LINC01237 | 0 | NHB | M |
| 8 | ICR_207 | chr3:50481168-50481455 | chr3:50481297-50481636 | CACNA2D2 | 0 | NHB | P |
| 9 | ICR_244 | chr4:3702565-3703061 | chr4:3703028-3703124 | LINC02171 | 24710 | ALL | P |
| 10 | ICR_281 | chr4:152009424-152009915 | chr4:152009643-152010206 | RNA5SP169 | 37811 | ALL | P |
| 11 | ICR_324 | chr5:110894251-110894443 | chr5:110894333-110894361 | BCLAF1P1 | 51830 | NHB | M |
| 12 | ICR_326*^ | chr5:136079156-136079563 | chr5:136079562-136079640 | TGFBI | 15338 | NHB | M |
| 13 | ICR_352 | chr5:171319025-171319892 | chr5:171318815-171319422 | TLX3 | 6886 | NHB | P |
| 14 | ICR_439*^ | chr7:5144439-5144757 | chr7:5144438-5144493 | ZNF890P | 0 | NHB | M |
| 15 | ICR_452^ | chr7:45564015-45564354 | chr7:45563920-45564036 | ADCY1 | 9786 | NHB | P |
| 16 | ICR_481*^# (A) | chr7:130490640-130494200 | chr7: 130494195- 130494648 | MEST\|MESTIT1 | 0 | NHB | M |
|  | ICR_481*^# (B) | chr7:130490640-130494200 | chr7: 130492063- 130492131 | MEST \| MESTIT1 | 0 | NHW | M |
|  | ICR_481*^# (C) | chr7:130490640-130494200 | chr7: 130492246- 130492270 | MEST \| MESTIT1 | 0 | ALL | M |
|  | ICR_481*^# (D) | chr7:130490640-130494200 | chr7: 130494195- 130494648 | MEST \| MESTIT1 | 0 | ALL | M |
| 17 | ICR_491 | chr7:155071148-155071376 | chr7:155071182-155071231 | HTR5A | 0 | NHB | M |
| 18 | ICR_548*^# | chr8:140098048-140100981 | chr8:140098530-140098591 | TRAPPC9 \| PEG13 | 0 | NHB | M |
| 19 | ICR_600 | chr9:87944657-87944766 | chr9:87944482-87944658 | SPATA31C1 | 21000 | NHB | M |
| 20 | ICR_633^ | chr10:5645451-5645631 | chr10:5645450-5645555 | ASB13 | 0 | NHB\|ALL | P |
| 21 | ICR_644^ | chr10:28326170-28327001 | chr10:28326690-28326841 | ZNF101P1 | 12276 | NHB | P |
| 22 | ICR_664^ | chr10:71266448-71266685 | chr10:71266523-71267095 | UNC5B | 0 | NHB | P |
| 23 | ICR_716*^#ε | chr11:1997886-1999417 | chr11:1998628-1998714 | MRPL23\|H19 | 0 | NHB | P |
| 24 | ICR_719*^#ε | chr11:2001655-2003118 | chr11:2002634-2002682 | MRPL23 | 0 | NHW\|ALL | P |
| 25 | ICR_814^&^ | chr13:20142811-20142911 | chr13:20142817-20142897 | GJA3 | 0 | NHB | M |
| 26 | ICR_827*^ | chr13:60267612-60268519 | chr13:60267683-60267898 | LINC00434 | 0 | ALL | M |
| 27 | ICR_829^ | chr13:80654682-80655272 | chr13:80654681-80654939 | PWWP2AP1 | 125 | NHB | M |
| 28 | ICR_893*^^&^ | chr15:24954592-24956828 | chr15:24955432-24955502 | SNHG1 \| SNRPN \| SNURF | 0 | NHB | M |
| 29 | ICR_914^ | chr15:99476322-99476786 | chr15:99476061-99476586 | LINC02244 | 73869 | NHB | P |
| 30 | ICR_1027 (A) | chr17:79517963-79518428 | chr17: 79517720- 79517977 | RBFOX3 | 0 | NHB | P |
|  | ICR_1027 (B) | chr17:79517963-79518428 | chr17 :79518326- 79518634 | RBFOX3 | 0 | NHB | P |
|  | ICR_1027 (C) | chr17:79517963-79518428 | chr17 79517916 79517973 | RBFOX3 | 0 | ALL | P |
| 31 | ICR_1079 | chr19:6509209-6509630 | chr19:6509440-6509508 | TUBB4A | 6361 | NHB | P |
| 32 | ICR_1142*^# | chr19:56837320-56841439 | chr19:56839300-56839440 | ZIM2 \| PEG3 \| MIMT1 | 0 | ALL | M |
| 33 | ICR_1191*^ | chr20:31547027-31548129 | chr20:31547408-31547421 | HM13 \| MCTS2P | 0 | ALL | M |
| 34 | ICR_1192^# (A) | chr20:37520202-37521842 | chr20: 37520201- 37520271 | BLCAP \| NNAT | 0 | NHB | M |
|  | ICR_1192^# (B) | chr20:37520202-37521842 | chr20: 37520954 -37521054 | BLCAP \| NNAT | 0 | NHB | M |
| 35 | ICR_1206*^#ε | chr20:58850158-58852357 | chr20:58851318-58851371 | GNAS | 0 | NHB | M |
| 36 | ICR_1207*^ε (A) | chr20:58853850-58856828 | chr20: 58855056- 58855067 | GNAS | 0 | NHB | M |
|  | ICR_1207*^ε (B) | chr20:58853850-58856828 | chr20: 58854476- 58854553 | GNAS | 0 | ALL | M |
| 37 | ICR_1377*^ | chr22:42532792-42533280 | chr22:42532982-42533635 | RRP7A | 12996 | NHB | P |

ICRs when the DMRs between AD cases and controls differed by ≥ 10% (Black) and ≥ 15% (Red)

* ICRs overlapping ENCODE annotated regions of CTCF binding (35)

^ ICRs overlapping ENCODE annotated regions of DNase I hypersensitivity

# ICRs overlapping previously published ICRs of imprinted genes (35, 55-68)

& ICRs that overlap previously established regions of systemic interindividual variation (SIVs) (35,91)

% Parental methylation denotes whether the paternal (P, sperm) or maternal (M, oocyte) allele of an ICR is methylated based upon both sperm and oocyte methylation data (35)

ε ICR_716 and ICR_719 overlap with the same known ICR in close proximity to *H19* |*MRPL23*; ICR_1206 and ICR_1207 overlap with the same known ICR in close proximity to *GNAS*

**Supplementary Table S4. Functional and pathway analyses of the genes closest to**

**AD-associated ICRs in NHBs.**

| **Molecular and Cellular Functions** | | |
| --- | --- | --- |
| **Name** | **p-value** | **Number of Molecules** |
| Gene expression | 1.77E-03 – 8.02E-06 | 4 |
| Cell morphology | 5.30E-03 – 1.20E-04 | 5 |
| Cellular development | 5.30E-03 – 1.20E-04 | 14 |
| Cell signaling | 2.65E-03 – 5.86E-04 | 4 |
| Nucleic acid metabolism | 2.65E-03 – 5.86E-04 | 4 |
| **Physiological System Development and Function** | | |
| **Name** | **p-value** | **Number of Molecules** |
| Cardiovascular system development and function | 5.30E-03 – 6.12E-05 | 7 |
| Embryonic development | 5.30E-03 – 6.12E-05 | 12 |
| Hematological system development and function | 5.30E-03 – 6.12E-05 | 12 |
| Lymphoid tissue structure and development | 5.30E-03 – 6.12E-05 | 8 |
| Organ development | 5.30E-03 – 6.12E-05 | 12 |
| **Canonical Pathways** | | |
| **Name** | **p-value** | **Number of Molecules** |
| Cardiac $\beta$-adrenergic signaling | 1.17E-04 | 5/180 (2.8%) |
| cAMP-mediated signaling | 4.09E-04 | 5/236 (2.1%) |
| White adipose tissue browning pathway | 4.99E-04 | 4/138 (2.9%) |
| Netrin signaling | 9.32E-04 | 3/72 (4.2%) |
| Gap junction signaling* | 1.90E-03 | 4/198 (2.0%) |

**Supplementary Table S5. Functional and pathway analyses of the genes closest to**

**AD-associated ICRs in NHWs.**

| **Molecular and Cellular Functions** | | |
| --- | --- | --- |
| **Name** | **p-value** | **Number of Molecules** |
| Cell signaling | 3.38E-02 – 4.63E-04 | 3 |
| Cellular development | 4.09E-02 – 4.63E-04 | 1 |
| Cellular growth and proliferation | 4.09E-02 – 4.63E-04 | 2 |
| Nucleic acid metabolism | 2.97E-02 – 4.63E-04 | 1 |
| Small molecule biochemistry | 4.63E-02 – 4.63E-04 | 3 |
| **Physiological System Development and Function** | | |
| **Name** | **p-value** | **Number of Molecules** |
| Organismal development | 4.85E-02 – 1.96E-04 | 6 |
| Embryonic development | 4.58E-02 – 4.63E-04 | 5 |
| Endocrine system development and function | 4.09E-02 – 4.63E-04 | 2 |
| Organ development | 4.58E-02 – 4.63E-04 | 3 |
| Tissue morphology | 4.58E-02 – 4.63E-04 | 5 |
| **Canonical Pathways** | | |
| **Name** | **p-value** | **Number of Molecules** |
| White adipose tissue browning pathway | 1.78E-03 | 2/138 (1.4%) |
| Cellular effects of Sildenafil (Viagra) | 2.10E-03 | 2/150 (1.3%) |
| Dopamine-DARPP32 feedback in cAMP signaling | 3.20E-03 | 2/186 (1.1%) |
| Synaptic long-term depression | 3.62E-03 | 2/198 (1.0%) |
| Gap junction signaling* | 3.62E-03 | 2/198 (1.0%) |

**
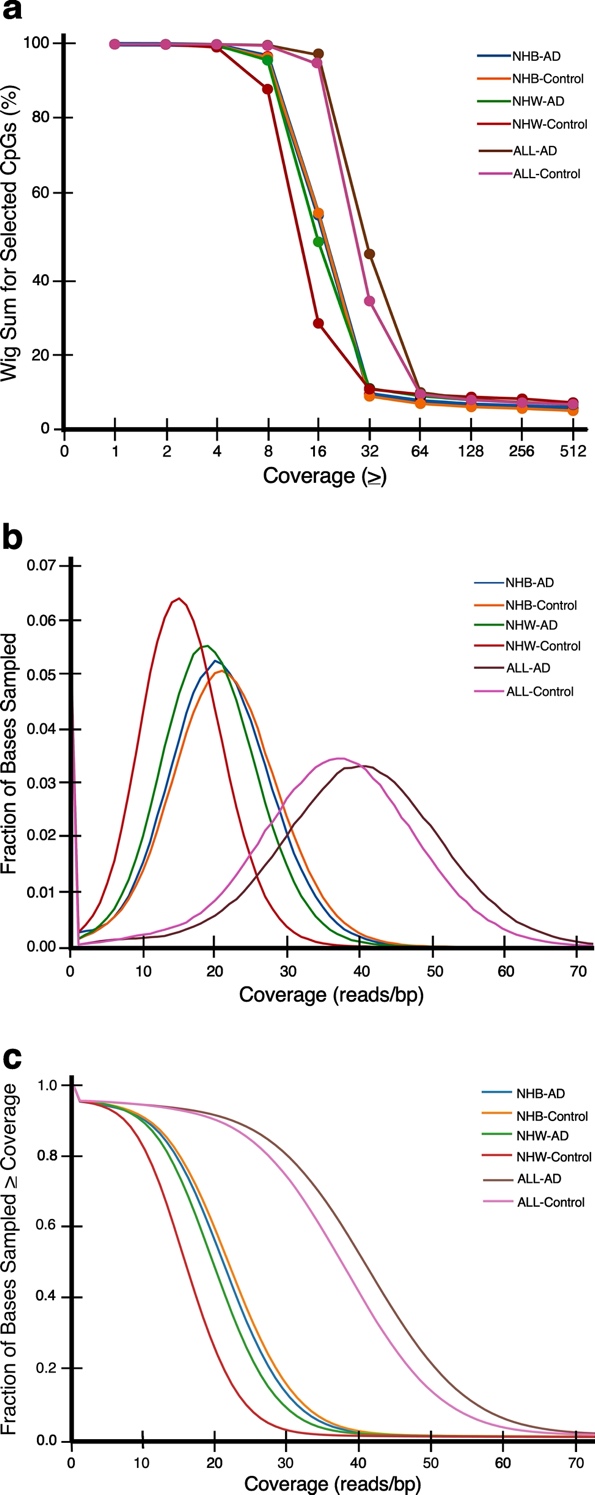
**

**Supplementary Fig. S1. Quality control of WGBS results.** **a** Duplication bias for DMRs between AD cases and controls calculated as the ratio of total coverage of DMR CpGs/total coverage of all CpGs, wig-sum percentage on y-axis at read depth $\geq$x. Random and unbiased sequencing is distinguished by wig sum percentage dropping below 10% past a certain read depth. **b** CpG coverage of bisulfite sequencing of NHB and NHW brain samples from AD cases and controls reported as fraction of bases with coverage = x, indicating mean and distribution, and **c** CpG coverage of bisulfite sequencing of NHB and NHW brain samples from AD cases and controls reported as fraction of bases with coverage $\geq$x.
